# Stolen childhood taking a toll at young adulthood: The higher risk of high blood pressure and high blood glucose comorbidity among child brides

**DOI:** 10.1371/journal.pgph.0000638

**Published:** 2022-06-24

**Authors:** Biplab Datta, Ashwini Tiwari, Lynn Glenn

**Affiliations:** 1 Institute of Public and Preventive Health, Augusta University, Augusta, GA, United States of America; 2 Department of Population Health Sciences, Medical College of Georgia, Augusta University, Augusta, GA, United States of America; 3 Department of Biobehavioral Nursing, College of Nursing, Augusta University, Augusta, GA, United States of America; Dow University of Health Sciences, PAKISTAN

## Abstract

Despite notable progress being made in preventing child marriage, a significant proportion of women worldwide are still married before reaching adulthood. Though many aspects of child marriage have been widely studied, little is known on the later life health outcomes of child brides, let alone the critical need for healthcare during adulthood. This paper examines whether child brides at a young adult age bear a greater risk of high blood pressure (HBP) and high blood glucose (HBG) comorbidity than those who were married as adults. Using nationally representative data from India, we categorized married young adult (aged 20-34 years) women in four categories: neither HBP nor HBG, HBP only, HBG only, and both HBP and HBG. We estimated multinomial logistic regressions to obtain unadjusted and adjusted relative risk ratios in favor of these mutually exclusive outcomes for the child marriage indicator. Around 0.5% of the women in our sample had high blood pressure and high blood glucose comorbidity. While the prevalence of comorbidity was 0.4% among women who were married as adults, comorbidity was 40% higher (*p* < 0.000) among women who were married as children. The relative risk of the comorbidity among child brides was 1.4 (95%*CI*: 1.2–1.7) times that of their peers who were not married as children. The findings, thus, suggest that child brides at young adult age are at greater risk of having high blood pressure and high blood glucose comorbidity. Concerted public health efforts, therefore, are necessary to improve their long-term health and wellbeing.

## Introduction

Girl child marriage is associated with a range of adverse consequences including higher risk of reproductive morbidity and mortality, lack of access to healthcare services, lower educational attainment and limited economic opportunities, higher risk of intimate partner violence, and lack of voice and agency [1, 2]. Though many aspects of child marriage have been widely studied in existing literature, there is a dearth of evidence on the later life health impacts of child marriage. Currently an estimated 650 million females around the world were married as children and the number could further increase by 170 million by 2030 if adequate measures are not taken [3]. Child brides thus constitute a significant share of currently living adult women, whose long-term health and wellbeing are at stake.

Child marriage is a key driver of adolescent childbearing that can impact long-term health outcomes of women [4]. Several studies document the association between adolescent childbearing and later life risks of hypertension and cardiovascular diseases [5, 6]. A couple of recent studies also report a higher risk of hypertension and other self-reported chronic conditions being associated with child marriage [7, 8]. Hypertension is often associated with diabetes mellitus comorbidity [9, 10]. Specifically, diagnosis of hypertension is one of the most common co-existing conditions among individuals with elevated glycated hemoglobin (*HbA*1*c*) and *type* 2 diabetes [11, 12]. The coexistence of these two conditions poses a greater risk of life-threatening macrovascular complications [13]. With millions of currently living young adult women married as children and millions of girls at risk of child marriage, examining the risk of hypertension and diabetes or raised blood glucose comorbidity among child brides is of critical public health interest. Early diagnosis and intervention could play an important role in reducing future disease burden and improving population health. We explore the relationship between child marriage and comorbidity of raised blood pressure and raised blood glucose using data from India, where incidence of child marriage, despite recent progress, is markedly high and the burden of noncommunicable, chronic diseases is gradually rising.

One third of the world’s child brides (approximately 223 million) live in India [14]. Around 40% of the young adult women (age 20 to 34 years) in India were married as children, and nearly 80% of the child brides in this age group entered motherhood at an adolescent age [15]. The prevalence of raised blood pressure and raised blood glucose among adult (age 18+) women in India was estimated 27.0% and 10.2% respectively [16]. In a sub-national survey, the prevalence of comorbid diabetes and hypertension was estimated at 1.5% among adult (age 18+) Indian women [17]. While a socioeconomic gradient in incidence of child marriage is observed in India [18], socioeconomic status conditions (SES) such as educational attainment have not demonstrated as significant predictors of hypertension and diabetes comorbidity [17]. A positive socioeconomic gradient, however, is observed for standalone diabetes and hypertension morbidities [19]; although there exist notable variations in the relationship between diabetes prevalence and low SES conditions across administrative regions in India [20].

As child brides experience various sociodemographic disadvantages in India, examining their risk of having hypertension and diabetes comorbidity entails a novel and relevant angle for prevention and control strategies. Though the prevalence and disparities in comorbid hypertension and diabetes among reproductive age women has been studied in some developed [21] and developing [22] countries, to our knowledge no studies to date explore the association between child marriage and hypertension and diabetes comorbidity. Understanding this association is important since identifying the at-risk population is critical for strategizing efficient public health interventions, particularly in low resource settings. This study, therefore, aims to examine whether the prevalence of high blood pressure (HBP) and high blood glucose (HBG) comorbidity in young adult women is higher among those who were married as children in India compared to their peers who were married as adults.

## Materials and methods

### Ethics statement

We used publicly available anonymized secondary data for our analysis. Participation in the survey was voluntary and informed consent was obtained prior interview. The survey protocol was approved by the International Institute for Population Sciences (IIPS) Institutional Review Board and ICF Institutional Review Board [15].

### Data

We obtained data on 262,205 married women of young adult age (20 to 34 years) from the 2015–16 wave of the India National Family Health Survey (NFHS-4). The NFHS-4 was conducted using a two-stage sampling framework and covers urban and rural areas of all 36 states (including union territories) of India. The NFHS-4 collects a variety of sociodemographic and anthropometric information form a nationally representative sample of women with a response rate of 97 percent.

### Measures

In the NFHS-4, respondent’s blood pressure was measured three times during a single visit with at least five minutes interval between each measure. Respondents were also asked if they were taking any medication to control HBP. An individual was identified as having HBP if the average systolic blood pressure (SBP) was greater than or equal to 140*mmHg* or if the average diastolic blood pressure (DBP) was greater than or equal to 90*mmHg* or if the individual was taking anti-hypertensive medication at the time of the survey [15].

Respondent’s random blood glucose level was also measured in the NFHS-4. An individual was identified as having HBG if the random blood glucose level was 141*mg*/*dl* or more [15]. Based on the blood pressure and blood glucose measures, individuals were categorized in four mutually exclusive categories: no HBP or HBG, HBP only, HBG only, and both HBP and HBG.

The NFHS-4 further reports respondent’s age at first marriage for women who were married at the time of the survey, from which we identified whether an individual was married before the legal age of 18 years. Self-reported age at first marriage could be subject to misreporting. To mitigate any bias from probable misreporting, we used an alternative measure of child marriage, captured by adolescent motherhood. Child marriage is regarded as the major contributing factor of adolescent pregnancy and childbearing [23]. Since childbirth in India mostly occurs within marriage [24], adolescent motherhood, defined as giving birth by age 19 years, is most likely to follow marriage before age 18 years. The NFHS-4 collects detailed birth history for each respondent, from which we were able to calculate age of women at first birth. Using this information, we created a binary variable indicating if a respondent gave birth during her adolescence (i.e., ≤19 years) and used that as an alternative measure of child marriage.

### Statistical analysis

We first estimated the share of women across the four mutually exclusive HBP and HBG comorbidity categories by child marriage. We conducted Wald tests to examine the equality of the share across women married as adults and married as children. We then estimated a multinomial logistic regression to obtain relative risk ratios (RRR) of the following mutually exclusive outcomes—i) HBP only, ii) HBG only, and iii) both HBP and HBG, relative to the base outcome of “neither HBP nor HBG” as follows:

$$\begin{array}{c} P\left(Y_{i}=d\;|\;\text{CM}\right)=\frac{exp(\beta_{0d}+\gamma_{d}CM_{i})}{1+\sum_{k=2}^{4}exp\left(\beta_{0k}+\gamma_{k}CM_{i}\right)} \end{array}$$ (1)

The mutually exclusive outcomes are denoted by *d* in Eq 1. Our explanatory variable, *CM*_*i*_, is a binary variable indicating whether respondent *i* was married as a child (i.e., before age 18 years) or not. We then estimated a multivariable specification to obtain adjusted relative risk ratios (ARRR) as follows:

$$\begin{array}{c} P\left(Y_{i}=d\;|\;\text{CM},\;X\right)=\frac{exp(\beta_{0d}+\gamma_{d}CM_{i}+{x_{i}}^{\prime }\beta_{jd}+State)}{1+\sum_{k=2}^{4}exp\left(\beta_{0k}+\gamma_{k}CM_{i}+{x_{i}}^{\prime }\beta_{jk}+State_{k}\right)} \end{array}$$ (2)

In this specification, **X** is a vector of sociodemographic correlates that include: age group—20 to 22 (reference group), 23 to 25, 26 to 28, 29 to 31, and 32 to 34 years; educational attainment—no education (reference group), primary, secondary, and higher; household size—3 or less (reference group), 4 to 5, 6 to 8, and 9+; household wealth index quintiles—1^st^ quintile (poorest) (reference group), 2^nd^ quintile, 3^rd^ quintile, 4^th^ quintile, and 5^th^ quintile (least poor); religion—Hindu (reference group), Muslim, Christian, Sikh, Buddhist, and other; caste—not backward class (reference group), scheduled caste, scheduled tribe, other backward class; and place of residence—rural (reference group), and urban. These covariates are common in literature exploring the sociodemographic factors associated with hypertension and diabetes as well as with child marriage [17, 22, 25]. In addition to these sociodemographic correlates, to account for state level variations in policies and healthcare provisions, we further controlled for state fixed effects. Of note, the correlates were added in the model for the purpose of examining whether the relationship between child marriage and the HBP and HBG comorbidity persisted after controlling for relevant socioeconomic attributes.

Next, in addition to the sociodemographic covariates in vector **X**, we controlled for factors that may impact HBP and/or HBG conditions in the following specification:

$$\begin{array}{c} P\left(Y_{i}=d\;|\;\text{CM},\;X,\;R\right)=\frac{exp(\beta_{0d}+\gamma_{d}CM_{i}+{x_{i}}^{\prime }\beta_{jd}+{r_{i}}^{\prime }\delta_{jd}+State)}{1+\sum_{k=2}^{4}exp\left(\beta_{0k}+\gamma_{k}CM_{i}+{x_{i}}^{\prime }\beta_{jk}+{r_{i}}^{\prime }\delta_{jk}+State_{k}\right)} \end{array}$$ (3)

**R** in Eq 3 is a vector of factors that include: nutritional status—normal (BMI 18.5 *to* 24.9 *kg*/*m*^2^—reference group), thin (BMI less than 18.5 *kg*/*m*^2^), overweight (BMI 25.0 *to* 29.9 *kg*/*m*^2^), and obese (BMI greater than 30.0 *kg*/*m*^2^); parity or the number of children born—none (reference group), 1, 2, and 3+; tobacco or alcohol consumption, oral contraception use, and pregnancy status. We examined whether the key results (i.e., ARRR for the child marriage indicator) persisted after controlling for these factors.

Lastly, we estimated the regression models with the alternative measure of child marriage, that is adolescent motherhood. We replaced *CM*_*i*_ in Eqs 1, 2 and 3 with *AM*_*i*_, a binary variable indicating whether respondent *i* gave birth during adolescent age. All estimates accounted for complex survey weights and analyses were conducted using Stata 17.0 software.

## Results

Around 40% of the young adult women in our sample were married before their 18^th^ birthday. Table 1 presents the background characteristics of study participants. The prevalence of child marriage was higher in rural areas than in urban areas. Across household wealth categories, the prevalence was the highest among the poorest quintile and the lowest among the richest quintile. Child marriage was more prevalent among scheduled castes and scheduled tribes. Child brides were less likely to attain higher education than their peers. Child brides, on the other hand, were more likely to consume tobacco or alcohol and give birth of two or more children.

**Table 1:** Background characteristics of the study participants by child marriage.

	All (%)	Married as adults (%)	Married as children (%)
Age group			
20–22	16.45	16.29	16.69
	[0.11], (16.24, 16.67)	[0.14], (16.02, 16.57)	[0.18], (16.33, 17.05)
23–25	22.31	23.89	20.02
	[0.12], (22.07, 22.55)	[0.16], (23.57, 24.22)	[0.18], (19.66, 20.37)
26–28	22.29	22.9	21.39
	[0.12], (22.05, 22.53)	[0.16], (22.59, 23.22)	[0.19], (21.03, 21.76)
29–31	21.01	20.02	22.45
	[0.12], (20.78, 21.25)	[0.15], (19.72, 20.33)	[0.19], (22.08, 22.82)
32–34	17.93	16.88	19.45
	[0.11], (17.71, 18.15)	[0.15], (16.59, 17.18)	[0.18], (19.10, 19.80)
Education			
No education	24.8	17.19	35.84
	[0.15], (24.49, 25.10)	[0.15], (16.90, 17.49)	[0.24], (35.38, 36.31)
Primary	13.99	10.64	18.86
	[0.11], (13.78, 14.20)	[0.11], (10.42, 10.87)	[0.19], (18.49, 19.23)
Secondary	48.6	52.61	42.78
	[0.18], (48.24, 48.96)	[0.23], (52.16, 53.06)	[0.25], (42.30, 43.27)
Higher	12.61	19.56	2.52
	[0.14], (12.33, 12.89)	[0.21], (19.14, 19.97)	[0.10], (2.32, 2.72)
Household size			
3 or less	12.69	14.63	9.87
	[0.12], (12.46, 12.92)	[0.16], (14.32, 14.94)	[0.15], (9.58, 10.16)
4–5	41.21	39.34	43.93
	[0.16], (40.90, 41.52)	[0.20], (38.95, 39.74)	[0.24], (43.46, 44.39)
6–8	30.95	30.46	31.67
	[0.16], (30.64, 31.26)	[0.21], (30.06, 30.87)	[0.21], (31.26, 32.08)
9+	15.15	15.56	14.54
	[0.14], (14.87, 15.42)	[0.17], (15.24, 15.89)	[0.19], (14.17, 14.91)
Wealth index quintiles			
1st quintile (poorest)	19	14.27	25.86
	[0.15], (18.70, 19.29)	[0.14], (13.99, 14.55)	[0.23], (25.42, 26.31)
2nd quintile	20.28	16.96	25.12
	[0.14], (20.01, 20.56)	[0.15], (16.66, 17.26)	[0.22], (24.70, 25.55)
3rd quintile	21.11	20.08	22.6
	[0.15], (20.82, 21.40)	[0.17], (19.74, 20.43)	[0.22], (22.18, 23.03)
4th quintile	21.02	23.6	17.28
	[0.17], (20.69, 21.36)	[0.21], (23.20, 24.01)	[0.22], (16.85, 17.71)
5th (least poor)	18.58	25.09	9.14
	[0.19], (18.21, 18.96)	[0.25], (24.60, 25.57)	[0.19], (8.77, 9.50)
Religion			
Hindu	81.2	80.69	81.94
	[0.24], (80.72, 81.67)	[0.27], (80.16, 81.22)	[0.31], (81.34, 82.54)
Muslim	13.66	13.17	14.37
	[0.23], (13.22, 14.11)	[0.25], (12.68, 13.66)	[0.29], (13.81, 14.93)
Christian	1.99	2.34	1.47
	[0.06], (1.87, 2.11)	[0.08], (2.18, 2.50)	[0.07], (1.33, 1.62)
Sikh	1.54	2.14	0.67
	[0.04], (1.46, 1.62)	[0.05], (2.04, 2.25)	[0.04], (0.59, 0.74)
Buddhist	0.88	1	0.7
	[0.06], (0.76, 1.00)	[0.08], (0.85, 1.16)	[0.06], (0.59, 0.81)
Other	0.74	0.66	0.85
	[0.07], (0.59, 0.88)	[0.07], (0.52, 0.79)	[0.11], (0.64, 1.06)
Caste			
Not backward class	25.67	27.66	22.78
	[0.22], (25.23, 26.10)	[0.27], (27.14, 28.18)	[0.29], (22.21, 23.34)
Scheduled caste	20.88	19.56	22.8
	[0.21], (20.46, 21.30)	[0.24], (19.08, 20.03)	[0.28], (22.25, 23.36)
Scheduled tribe	9.57	8.49	11.14
	[0.14], (9.30, 9.84)	[0.14], (8.23, 8.76)	[0.19], (10.76, 11.52)
Other backward class	43.88	44.29	43.28
	[0.24], (43.41, 44.35)	[0.28], (43.74, 44.84)	[0.30], (42.70, 43.87)
Residence			
Rural	68.32	63.38	75.5
	[0.25], (67.84, 68.81)	[0.30], (62.80, 63.96)	[0.29], (74.94, 76.06)
Urban	31.68	36.62	24.5
	[0.25], (31.19, 32.16)	[0.30], (36.04, 37.20)	[0.29], (23.94, 25.06)
Obs.	262205	160505	101700

Estimates were obtained using complex survey weights. Linearized standard errors are in square brackets. 95% confidence intervals are in parenthesis. Shares add to 100 across rows for respective characteristics.

The mean SBP and DBP in the sample was 112 *mmHg* and 75 *mmHg* respectively, and the mean blood glucose level was 101 *mg*/*dl*. Around 6% of the women in the sample had HBP and nearly 4% had HBG. 91.2% of the women, who were not married as children, neither had HBP nor HBG. The prevalence of “not having HBP or HBG” was 1.6 percentage points lower (*p* < 0.000) among those who were married before age 18 years. The prevalence of “HBP only” was higher (*p* < 0.000) among child brides and there was not much difference in the prevalence of “HBG only” among child brides and those who were married as adults. While 0.4% of the women who were married as adults (at or after age 18 years) had both HBP and HBG, the share of HBP and HBG comorbidity was 40% higher (*p* < 0.000) among women who were married as children (Table 2).

**Table 2:** Share of young adult women having blood pressure and blood glucose conditions by child marriage.

	All (%)	Married as adults (%)	Married as children (%)
Neither high blood pressure nor blood glucose	90.55	91.21	89.58^†^
	[0.09]	[0.11]	[0.14]
	(90.37, 90.72)	(90.99, 91.43)	(89.30, 89.86)
High blood pressure only	5.61	5.06	6.41^†^
	[0.07]	[0.09]	[0.11]
	(5.47, 5.75)	(4.89, 5.23)	(6.19, 6.63)
High blood glucose only	3.38	3.33	3.46
	[0.06]	[0.08]	[0.08]
	(3.27, 3.49)	(3.18, 3.48)	(3.30, 3.62)
Both high blood pressure & blood glucose	0.46	0.4	0.56^†^
	[0.02]	[0.02]	[0.03]
	(0.43, 0.50)	(0.35, 0.45)	(0.49, 0.62)
Obs.	262205	160505	101700

Estimates were obtained using complex survey weights. Linearized standard errors are in square brackets. 95% confidence intervals are in parenthesis.

^†^ refers share for child marriage group significantly different (*p* < 0.000) from share for no child marriage group.

Panel A in Table 3 presents the RRRs in favor of the mutually exclusive HBP and HBG outcomes associated with child marriage. Relative to the “neither HBP nor HBG” outcome, the relative risk of the “HBP and HBG comorbidity” for women who were married as children were 1.4 times that of those who were married as adults. The relative risks of “HBP only” and “HBG only” outcomes were also greater for child brides compared to their peers.

**Table 3:** Unadjusted and adjusted relative risk ratios for child marriage indicator.

	Base outcome: Neither HBP nor HBG	Outcome 1: HBP only	Outcome 2: HBG only	Outcome 3: Both HBP & HBG
**A. Unadjusted**				
Child marriage		1.291***	1.057	1.407***
		(1.228, 1.357)	(0.990, 1.130)	(1.189, 1.666)
**B. Adjusted for sociodemographic correlates**				
Child marriage		1.250***	1.074**	1.464***
		(1.185, 1.318)	(1.003, 1.149)	(1.229, 1.743)
**C. Adjusted sociodemographic correlates correlates & risk factors**				
Child marriage		1.229***	1.079**	1.464***
		(1.163, 1.298)	(1.006, 1.158)	(1.221, 1.754)

Estimates were obtained using complex survey weights.

***p < 0.01,

**p < 0.05.

95% confidence intervals are in parenthesis. Panels A, B, and C respectively present results of the multinomial logistic regression specifications in Eqs 1, 2, and 3. Sociodemographic covariates (not reported here) include age, educational attainment, household size, wealth index quintiles, religion, caste, urban/rural residence. Risk factors (not reported here) include parity, body mass index categories, tobacco/alcohol consumption, and current pregnancy status. Both multivariable specifications account for state fixed effects.

The ARRRs for child marriage, adjusted for sociodemographic correlates, are presented in Panel B in Table 3. The adjusted relative risks associated with child marriage for all three outcomes were very similar to the unadjusted relative risks reported in Panel A. Relative to the base outcome, a child bride’s adjusted relative risk of having HBP and HBG comorbidity were 1.5 times that of those who were married as adults. The ARRRs for child marriage, across all three outcomes, adjusted of sociodemographic correlates and risk factors, persisted when we account for respondent’s nutritional status, current pregnancy status, oral contraception use, tobacco or alcohol consumption and number of children born (Panel C of Table 3).

Results for the adolescent motherhood, the alternative measure of child marriage, are presented in Table 4. The correlation between age at first birth and age at first marriage in our sample was 0.83. The tetrachoric correlation between the indicator variables of child marriage and adolescent motherhood was 0.88—suggesting a strong relationship between the two measures. Adolescent mothers’ adjusted relative risk of HBP and HBG comorbidity was 1.3 to 1.4 times that of those who did not give birth during adolescence. These results further reinforced the relationship between child marriage and HBP and HBG comorbidity among young adult women in India.

**Table 4:** Unadjusted and adjusted relative risk ratios for adolescent motherhood indicator.

	Base outcome: Neither HBP nor HBG	Outcome 1: HBP only	Outcome 2: HBG only	Outcome 3: Both HBP & HBG
**A. Unadjusted**				
Adolescent motherhood		1.230***	1.064	1.295***
		(1.171, 1.293)	(0.992, 1.141)	(1.091, 1.536)
**B. Adjusted for sociodemographic correlates**				
Adolescent motherhood		1.203***	1.067	1.337***
		(1.141, 1.267)	(0.992, 1.149)	(1.123, 1.590)
**C. Adjusted sociodemographic correlates & risk factors**				
Adolescent motherhood		1.212***	1.103**	1.401***
		(1.146, 1.283)	(1.018, 1.196)	(1.165, 1.685)

Estimates were obtained using complex survey weights.

***p < 0.01,

**p < 0.05.

95% confidence intervals are in parenthesis. Panels A, B, and C respectively present results of the multinomial logistic regression specifications in Eqs 1, 2, and 3 with *CM* replaced with *AM*. Sociodemographic covariates (not reported here) include age, educational attainment, household size, wealth index quintiles, religion, caste, urban/rural residence. Risk factors (not reported here) include parity, body mass index categories, tobacco/alcohol consumption, and current pregnancy status. Both multivariable specifications account for state fixed effects.

## Discussion

Child brides represent a marginalized and vulnerable segment of the global population susceptible to various forms of socioeconomic deprivations in many developing countries. The physical and mental wellbeing of child brides at adult age are often subject to societal negligence, partly because of lack of evidence on the long-term health consequences of child marriage. In this paper, we document a disproportionately higher risk of hypertension and HBG comorbidity among child brides at young adulthood, compared to their peers married as adults. After accounting for household wealth, education, and other sociodemographic correlates as well as observed risk factors for hypertension and diabetes, our results demonstrate a robust relationship between child marriage and comorbidity of HBP and HBG.

There is a growing body of literature that highlights how exposure to physical, psychosocial, and environmental factors over the life course plays critical role in shaping current health outcomes [26]. Studies document relationships between chronic conditions, such as cardiovascular diseases and *type* 2 diabetes, and early life process during childhood and adolescence [27]. Child marriage forces a girl to play adult roles before reaching adulthood, which could have detrimental physical and psychological consequences on health across the life course. Our findings that child brides bearing a higher risk of HBP and HBG comorbidity compared to their peers add to the growing body of literature exploring the life course approaches to chronic diseases.

Of importance, associations between pre-existing diabetes and hypertension and poor maternal health outcomes are well established, such as a heightened risk of congenital anomalies, fetal demise, preterm births, perinatal mortality, and pre-eclampsia [28]. Studies also document child marriage’s association with higher fertility, unwanted pregnancy, and pregnancy termination [25, 29]. The association between child marriage and HBP and HBG comorbidity, therefore, is intertwined with maternal health outcomes. Despite experiencing a dramatic decline in maternal mortality over the past decade, an estimated 45,000 maternal deaths occurred in India in 2015, which was around 19% of the global maternal deaths [30]. As such, the presentation of chronic disease among child brides may have significant ramifications for maternal health and well-being if not addressed via prevention or behavioral intervention efforts.

Undiagnosed diabetes and hypertension are critical public health concerns worldwide. High blood sugar or hyperglycemia awareness is considerably low in the Indian population contributing to suboptimal control rate [31]. Research also documents low awareness and control of hypertension or HBP in India [32]. Uncontrolled and untreated diabetes and hypertension can lead to an increased risk of cardiovascular diseases [33]. In this study, we identified a group of population (i.e., child brides) who are at heightened risk of HBP and HBG comorbidity. Therefore, targeted interventions for health promotion in this group could be an effective strategy for prevention and control of certain chronic conditions. In addition to the challenges associated with screening and diagnosis, providing adequate care for managing HBP and HBG is a challenge in low-and-middle income countries (LMICs). Treatment of these conditions in the LMICs is associated with catastrophic health expenditure and could disrupt household consumption of essential commodities [34, 35]. Since child marriage is linked to poverty and adverse economic consequences, the disease burden of HBP and HBG could further exacerbate the economic wellbeing of child brides. A concerted effort, therefore, is warranted to mitigate the risk of the HBP and HBG disease burden among child brides.

Our research highlights that social determinants of health warrants further consideration and research. In India, the prevalence of hypertension and diabetes is typically higher in urbanized areas and among more affluent social groups [32, 36]. However, we found that the odds of having HBP and HBG comorbidity were significantly higher among child brides even after controlling for socioeconomic attributes such as household income and educational attainment as well as rural or urban residence and state fixed effects. This finding indicates that irrespective of sociodemographic background, the inherent nature of being married at a young-age places these young brides at risk of later life health outcomes as a result.

The study is subject to some limitations. First, the HBP and HBG conditions were not clinically diagnosed. Fasting glucose measurements, for instance, would have been more accurate in determining HBG condition. However, unlike other studies where respondent’s self-reported hypertension and/or diabetes conditions are used as prevalence measures [22], we used measured blood pressure and blood glucose levels to determine comorbidity. Second, age at first marriage data were self-reported. To mitigate any bias from probable misreporting, we used an alternative measure of child marriage that exploits the strong relationship between child marriage and adolescent motherhood. Lastly, because of the cross-sectional nature of data, we could not investigate the casual pathways of the relationship between child marriage and HBP and HBG comorbidity at young adult age. Future research using longitudinal data will shed more light on this issue.

## Conclusion

Our findings suggest that child brides at young adult age are more susceptible to HBP and HBG comorbidity. Our analyses can be linked to at least two of the United Nations Sustainable Development Goal (SDG) targets—target 3.4: reducing premature mortality from non-communicable diseases and target 5.4: eliminating child marriage [37]. Greater efforts are to eliminate the practice of child marriage as it has consequences for later life health outcomes. Orchestrated public health efforts to eliminate child marriage and to provide access to necessary healthcare, therefore, will improve the health and wellbeing of women in the developing countries as well as will facilitate attaining certain UN SDG targets. Further, targeted prevention and control initiatives are needed for the child brides who bear a disproportionately higher health risk than their peers married as adults.

## Supporting information

S1 Table Adjusted relative risk ratios in favor of mutually exclusive high blood pressure and high blood glucose outcomes for child marriage and sociodemographic correlates. Estimates were obtained using complex survey weights. ***p < 0.01, **p < 0.05. 95% confidence intervals are in parenthesis.
(DOCX)

S2 Table Adjusted relative risk ratios in favor of mutually exclusive high blood pressure and high blood glucose outcomes for child marriage, sociodemographic correlates, and hypertension and diabetes risk factors. Estimates were obtained using complex survey weights. ***p < 0.01, **p < 0.05. 95% confidence intervals are in parenthesis.
(DOCX)

S3 Table Adjusted relative risk ratios in favor of mutually exclusive high blood pressure and high blood glucose outcomes for adolescent motherhood and sociodemographic correlates. Estimates were obtained using complex survey weights. ***p < 0.01, **p < 0.05. 95% confidence intervals are in parenthesis.
(DOCX)

S4 Table Adjusted relative risk ratios in favor of mutually exclusive high blood pressure and high blood glucose outcomes for adolescent motherhood, sociodemographic correlates, and hypertension and diabetes risk factors. Estimates were obtained using complex survey weights. ***p < 0.01, **p < 0.05. 95% confidence intervals are in parenthesis.
(DOCX)

## References

[1] Mathur S, Greene M, Malhotra A. Too young to wed: the lives, rights and health of young married girls. Washington DC: International Center for Research on Women; 2003. Available from: https://www.issuelab.org/resources/11421/11421.pdf.

[2] Parsons J, Edmeades J, Kes A, Petroni S, Sexton M, Wodon Q. Economic impacts of child marriage: a review of the literature. The Review of Faith & International Affairs. 2015;13(3):12–22. doi: 10.1080/15570274.2015.1075757

[3] United Nation Children’s Fund (UNICEF). Looking Ahead Towards 2030: Eliminating child marriage through a decade of action; 2020. Available from: https://data.unicef.org/resources/looking-ahead-towards-2030-eliminating-child-marriage-through-a-decade-of-action/.

[4] Patel PH, Sen B. Teen motherhood and long-term health consequences. Maternal and child health journal. 2012;16(5):1063–1071. doi: 10.1007/s10995-011-0829-2 21656056

[5] Datta BK, Husain MJ, Kostova D. Hypertension in women: the role of adolescent childbearing. BMC public health. 2021;21(1):1–14. doi: 10.1186/s12889-021-11488-z 34325686 PMC8323295

[6] Rosendaal NT, Alvarado B, Wu YY, Velez MP, da Câmara SMA, Pirkle CM. Adolescent childbirth is associated with greater Framingham risk scores for cardiovascular disease among participants of the IMIAS (international mobility in aging study). Journal of the American Heart Association. 2017;6(11):e007058. doi: 10.1161/JAHA.117.007058 29092844 PMC5721784

[7] Datta B, Tiwari A. Adding to her woes: child bride’s higher risk of hypertension at young adulthood. Journal of Public Health. 2022;. doi: 10.1093/pubmed/fdac026 35257178

[8] Vikram K. Early marriage and health among women at midlife: Evidence from India. Journal of Marriage and Family. 2021;83(5):1480–1501. doi: 10.1111/jomf.12793

[9] Emdin CA, Anderson SG, Woodward M, Rahimi K. Usual blood pressure and risk of new-onset diabetes: evidence from 4.1 million adults and a meta-analysis of prospective studies. Journal of the American College of Cardiology. 2015;66(14):1552–1562. doi: 10.1016/j.jacc.2015.07.059 26429079 PMC4595710

[10] Tsimihodimos V, Gonzalez-Villalpando C, Meigs JB, Ferrannini E. Hypertension and diabetes mellitus: coprediction and time trajectories. Hypertension. 2018;71(3):422–428. doi: 10.1161/HYPERTENSIONAHA.117.10546 29335249 PMC5877818

[11] Bower JK, Appel LJ, Matsushita K, Young JH, Alonso A, Brancati FL, et al. Glycated hemoglobin and risk of hypertension in the atherosclerosis risk in communities study. Diabetes care. 2012;35(5):1031–1037. doi: 10.2337/dc11-2248 22432110 PMC3329825

[12] Iglay K, Hannachi H, Joseph Howie P, Xu J, Li X, Engel SS, et al. Prevalence and co-prevalence of comorbidities among patients with type 2 diabetes mellitus. Current medical research and opinion. 2016;32(7):1243–1252. doi: 10.1185/03007995.2016.1168291 26986190

[13] Bretzel RG. Comorbidity of diabetes mellitus and hypertension in the clinical setting: a review of prevalence, pathophysiology, and treatment perspectives. Clinical therapeutics. 2007;29:S35–S43. doi: 10.1016/j.clinthera.2007.07.010

[14] United Nation Children’s Fund (UNICEF). Ending Child Marriage: A profile of progress in India; 2019. Available from: https://data.unicef.org/resources/ending-child-marriage-a-profile-of-progress-in-india/.

[15] International Institute for Population Sciences (IIPS) and ICF. National Family Health Survey (NFHS-4), 2015–16: India; 2017. Available from: https://dhsprogram.com/pubs/pdf/FR339/FR339.pdf

[16] Mathur P, Kulothungan V, Leburu S, Krishnan A, Chaturvedi HK, Salve HR, et al. National noncommunicable disease monitoring survey (NNMS) in India: Estimating risk factor prevalence in adult population. PloS one. 2021;16(3):e0246712. doi: 10.1371/journal.pone.0246712 33651825 PMC7924800

[17] Tripathy JP, Thakur J, Jeet G, Jain S. Prevalence and determinants of comorbid diabetes and hypertension: Evidence from non communicable disease risk factor STEPS survey, India. Diabetes & Metabolic Syndrome: Clinical Research & Reviews. 2017;11:S459–S465. doi: 10.1016/j.dsx.2017.03.036 28395951

[18] Mishra T, Banerjee T, et al. Child marriage: some facts from selected Indian states”. Economics Bulletin. 2020;40(3):2093–2110.

[19] Corsi DJ, Subramanian S. Socioeconomic gradients and distribution of diabetes, hypertension, and obesity in India. JAMA network open. 2019;2(4):e190411–e190411. doi: 10.1001/jamanetworkopen.2019.0411 30951154 PMC6450330

[20] Anjana RM, Deepa M, Pradeepa R, Mahanta J, Narain K, Das HK, et al. Prevalence of diabetes and prediabetes in 15 states of India: results from the ICMR–INDIAB population-based cross-sectional study. The lancet Diabetes & endocrinology. 2017;5(8):585–596. doi: 10.1016/S2213-8587(17)30174-2 28601585

[21] Britton LE, Berry DC, Hussey JM. Comorbid hypertension and diabetes among US women of reproductive age: Prevalence and disparities. Journal of Diabetes and its Complications. 2018;32(12):1148–1152. doi: 10.1016/j.jdiacomp.2018.09.014 30291018 PMC6289742

[22] Yaya S, El-Khatib Z, Ahinkorah BO, Budu E, Bishwajit G. Prevalence and Socioeconomic Factors of Diabetes and High Blood Pressure Among Women in Kenya: A Cross-Sectional Study. Journal of Epidemiology and Global Health. 2021;11(4):397–404. doi: 10.1007/s44197-021-00004-6 34734380 PMC8664325

[23] United Nations Population Fund (UNFPA). Girlhood, Not Motherhood: Preventing Adolescent Pregnancy; 2015. Available from: https://www.unfpa.org/publications/girlhood-not-motherhood

[24] Ibarra-Nava I, Choudhry V, Agardh A. Desire to delay the first childbirth among young, married women in India: a cross-sectional study based on national survey data. BMC Public Health. 2020;20(1):1–10. doi: 10.1186/s12889-020-8402-9 32183765 PMC7079505

[25] Anita R, Saggurti N, Balaiah D, Silverman G. Prevalence of Child Marriage and its Impact on the Fertility and Fertility Control Behaviors of Young Women in India. Lancet. 2010;373(9678):1883–9. 10.1016/S0140-6736(09)60246-4 PMC2759702 19278721

[26] Jones NL, Gilman SE, Cheng TL, Drury SS, Hill CV, Geronimus AT. Life course approaches to the causes of health disparities. American journal of public health. 2019;109(S1):S48–S55. doi: 10.2105/AJPH.2018.304738 30699022 PMC6356123

[27] Lynch J, Smith GD. A life course approach to chronic disease epidemiology. Annu Rev Public Health. 2005;26:1–35. doi: 10.1146/annurev.publhealth.26.021304.144505 15760279

[28] Sullivan SD, Umans JG, Ratner R. Hypertension complicating diabetic pregnancies: pathophysiology, management, and controversies. The Journal of Clinical Hypertension. 2011;13(4):275–284. doi: 10.1111/j.1751-7176.2011.00440.x 21466626 PMC8673181

[29] Kamal SM, Ulas E. Child marriage and its impact on fertility and fertility-related outcomes in South Asian countries. International Sociology. 2021; p. 0268580920961316. doi: 10.1177/0268580920961316

[30] World Health Organization (WHO). Trends in Maternal Mortality: 1990 to 2015 – Estimates by WHO, UNICEF, UNFPA, World Bank Group and the United Nations Population Division; 2015. Available from: https://www.unfpa.org/publications/trends-maternal-mortality-1990-2015.

[31] Devi P, Rao M, Sigamani A, Faruqui A, Jose M, Gupta R, et al. Prevalence, risk factors and awareness of hypertension in India: a systematic review. Journal of human hypertension. 2013;27(5):281–287. doi: 10.1038/jhh.2012.33 22971751

[32] Gupta R, Gaur K, Ram CVS. Emerging trends in hypertension epidemiology in India. Journal of human hypertension. 2019;33(8):575–587. doi: 10.1038/s41371-018-0117-3 30254382

[33] Petrie JR, Guzik TJ, Touyz RM. Diabetes, hypertension, and cardiovascular disease: clinical insights and vascular mechanisms. Canadian Journal of Cardiology. 2018;34(5):575–584. doi: 10.1016/j.cjca.2017.12.005 29459239 PMC5953551

[34] Datta BK, Husain MJ, Asma S. Assessing the relationship between out-of-pocket spending on blood pressure and diabetes medication and household catastrophic health expenditure: evidence from Pakistan. International journal for equity in health. 2019;18(1):1–12. doi: 10.1186/s12939-018-0906-x 30646905 PMC6334430

[35] Datta BK, Husain MJ, Fatehin S. The crowding out effect of out-of-pocket medication expenses of two major non-communicable diseases in Pakistan. International health. 2020;12(1):50–59. doi: 10.1093/inthealth/ihz075 31608937 PMC11833703

[36] Tandon N, Anjana RM, Mohan V, Kaur T, Afshin A, Ong K, et al. The increasing burden of diabetes and variations among the states of India: the Global Burden of Disease Study 1990–2016. The Lancet Global Health. 2018;6(12):e1352–e1362. doi: 10.1016/S2214-109X(18)30387-5 30219315 PMC6227383

[37] 24 United Nations (UN). Transforming our World: The 2030 Agenda for Sustainable Development; 2015. Available from: https://sdgs.un.org/2030agenda.

