# Supplementary material for: Stolen childhood taking a toll at young adulthood: The higher risk of high blood pressure and high blood glucose comorbidity among child brides
Source: PLOS Glob Public Health. 2022 Jun 24;2(6):e0000638. doi: 10.1371/journal.pgph.0000638 (PMC10021810; doi:10.1371/journal.pgph.0000638)
Supplement: S1 Table — Estimates were obtained using complex survey weights. ***p < 0.01, **p < 0.05. 95% confidence intervals are in parenthesis. (DOCX) [file pgph.0000638.s001.docx]

**S1 Table**. Adjusted relative risk ratios in favor of mutually exclusive high blood pressure and high blood glucose outcomes for child marriage and sociodemographic correlates

|  | **Base outcome:** | **Outcome 1:** | **Outcome 2:** | **Outcome 3:** |
| --- | --- | --- | --- | --- |
|  | Neither High  Blood Pressure  nor Blood  Glucose | High  Blood  Pressure  only | High  Blood  Glucose  only | Both High  Blood Pressure  And Blood  Glucose |
|  |  |  |  |  |
| Child marriage |  | 1.250*** | 1.074** | 1.464*** |
|  |  | (1.185, 1.318) | (1.003, 1.149) | (1.229, 1.743) |
| Age group |  |  |  |  |
| *20-22* | Ref. |  |  |  |
| *23-25* |  | 1.424*** | 1.107 | 1.485 |
|  |  | (1.293, 1.569) | (0.980, 1.251) | (0.900, 2.450) |
| *26-28* |  | 1.930*** | 1.412*** | 3.262*** |
|  |  | (1.756, 2.121) | (1.258, 1.584) | (2.095, 5.079) |
| *29-31* |  | 2.661*** | 1.797*** | 5.365*** |
|  |  | (2.428, 2.918) | (1.592, 2.028) | (3.482, 8.266) |
| *32-34* |  | 3.584*** | 2.192*** | 8.281*** |
|  |  | (3.270, 3.928) | (1.962, 2.450) | (5.392, 12.720) |
| Education |  |  |  |  |
| *No education* | Ref. |  |  |  |
| *Primary* |  | 1.010 | 0.896** | 1.046 |
|  |  | (0.937, 1.090) | (0.807, 0.994) | (0.802, 1.363) |
| *Secondary* |  | 0.942 | 0.999 | 0.907 |
|  |  | (0.881, 1.006) | (0.914, 1.090) | (0.720, 1.143) |
| *Higher* |  | 0.783*** | 0.935 | 0.786 |
|  |  | (0.695, 0.883) | (0.813, 1.075) | (0.556, 1.113) |
| Household size |  |  |  |  |
| *3 or less* | Ref. |  |  |  |
| *4-5* |  | 0.842*** | 0.999 | 0.680*** |
|  |  | (0.775, 0.914) | (0.892, 1.119) | (0.530, 0.873) |
| *6-8* |  | 0.746*** | 0.937 | 0.620*** |
|  |  | (0.686, 0.812) | (0.834, 1.053) | (0.476, 0.807) |
| *9+* |  | 0.729*** | 0.970 | 0.683** |
|  |  | (0.658, 0.806) | (0.854, 1.102) | (0.502, 0.930) |
| Wealth index quintiles |  |  |  |  |
| *1^st^ (Poorest)* | Ref. |  |  |  |
| *2^nd^ (Poorer)* |  | 0.936 | 1.081 | 1.228 |
|  |  | (0.869, 1.008) | (0.977, 1.195) | (0.925, 1.631) |
| *3^rd^ (Middle)* |  | 0.995 | 1.183*** | 1.242 |
|  |  | (0.916, 1.082) | (1.060, 1.320) | (0.919, 1.677) |
| *4^th^ (Richer)* |  | 1.214*** | 1.491*** | 1.631*** |
|  |  | (1.110, 1.328) | (1.315, 1.690) | (1.175, 2.263) |
| *5^th^ (Richest)* |  | 1.174*** | 1.447*** | 1.879*** |
|  |  | (1.050, 1.314) | (1.251, 1.675) | (1.281, 2.756) |
| Religion |  |  |  |  |
| *Hindu* | Ref. |  |  |  |
| *Muslim* |  | 1.273*** | 1.181*** | 1.241 |
|  |  | (1.180, 1.374) | (1.057, 1.321) | (0.981, 1.570) |
| *Christian* |  | 1.085 | 1.371 | 2.106*** |
|  |  | (0.866, 1.358) | (0.978, 1.923) | (1.296, 3.422) |
| *Sikh* |  | 1.317*** | 0.791 | 0.619 |
|  |  | (1.100, 1.578) | (0.579, 1.079) | (0.345, 1.112) |
| *Buddhist* |  | 1.011 | 1.290 | 0.537 |
|  |  | (0.745, 1.374) | (0.789, 2.108) | (0.159, 1.815) |
| *Other* |  | 1.511** | 0.847 | 2.046 |
|  |  | (1.057, 2.161) | (0.544, 1.317) | (0.910, 4.598) |
| Caste |  |  |  |  |
| *Not backward class* | Ref. |  |  |  |
| *Scheduled caste* |  | 0.954 | 1.043 | 0.841 |
|  |  | (0.877, 1.037) | (0.931, 1.169) | (0.653, 1.082) |
| *Scheduled tribe* |  | 1.143*** | 1.069 | 1.110 |
|  |  | (1.040, 1.256) | (0.886, 1.290) | (0.819, 1.505) |
| *Other backward class* |  | 0.988 | 0.997 | 0.900 |
|  |  | (0.922, 1.058) | (0.913, 1.089) | (0.727, 1.114) |
| Residence |  |  |  |  |
| *Rural* | Ref. |  |  |  |
| *Urban* |  | 0.963 | 0.948 | 1.113 |
|  |  | (0.896, 1.034) | (0.867, 1.035) | (0.891, 1.390) |
|  |  |  |  |  |
| State Fixed Effect |  | Yes | Yes | Yes |
|  |  |  |  |  |

Note: Estimates were obtained using complex survey weights. *** p<0.01, ** p<0.05. 95% confidence intervals are in parenthesis.
