# Supplementary material for: Stolen childhood taking a toll at young adulthood: The higher risk of high blood pressure and high blood glucose comorbidity among child brides
Source: PLOS Glob Public Health. 2022 Jun 24;2(6):e0000638. doi: 10.1371/journal.pgph.0000638 (PMC10021810; doi:10.1371/journal.pgph.0000638)
Supplement: S2 Table — Estimates were obtained using complex survey weights. ***p < 0.01, **p < 0.05. 95% confidence intervals are in parenthesis. (DOCX) [file pgph.0000638.s002.docx]

**S2 Table**. Adjusted relative risk ratios in favor of mutually exclusive high blood pressure and high blood glucose outcomes for child marriage, sociodemographic correlates, and hypertension and diabetes risk factors

|  | **Base outcome:** | **Outcome 1:** | **Outcome 2:** | **Outcome 3:** |
| --- | --- | --- | --- | --- |
|  | Neither High  Blood Pressure  nor Blood  Glucose | High  Blood  Pressure  only | High  Blood  Glucose  only | Both High  Blood Pressure  And Blood  Glucose |
|  |  |  |  |  |
| Child marriage |  | 1.229*** | 1.079** | 1.464*** |
|  |  | (1.163, 1.298) | (1.006, 1.158) | (1.221, 1.754) |
| Age group |  |  |  |  |
| *20-22* | Ref. |  |  |  |
| *23-25* |  | 1.391*** | 1.128 | 1.446 |
|  |  | (1.260, 1.535) | (0.996, 1.279) | (0.867, 2.410) |
| *26-28* |  | 1.789*** | 1.415*** | 3.021*** |
|  |  | (1.622, 1.973) | (1.252, 1.599) | (1.910, 4.777) |
| *29-31* |  | 2.335*** | 1.743*** | 4.634*** |
|  |  | (2.113, 2.580) | (1.519, 1.999) | (2.931, 7.326) |
| *32-34* |  | 3.014*** | 2.070*** | 6.758*** |
|  |  | (2.723, 3.337) | (1.825, 2.348) | (4.267, 10.702) |
| Education |  |  |  |  |
| *No education* | Ref. |  |  |  |
| *Primary* |  | 0.974 | 0.881** | 0.968 |
|  |  | (0.903, 1.051) | (0.794, 0.977) | (0.739, 1.268) |
| *Secondary* |  | 0.881*** | 0.962 | 0.786* |
|  |  | (0.823, 0.943) | (0.881, 1.050) | (0.617, 1.002) |
| *Higher* |  | 0.727*** | 0.883 | 0.688** |
|  |  | (0.645, 0.819) | (0.766, 1.016) | (0.479, 0.988) |
| Household size |  |  |  |  |
| *3 or less* | Ref. |  |  |  |
| *4-5* |  | 0.899** | 1.059 | 0.817 |
|  |  | (0.825, 0.979) | (0.943, 1.188) | (0.628, 1.062) |
| *6-8* |  | 0.817*** | 1.005 | 0.780 |
|  |  | (0.747, 0.893) | (0.888, 1.137) | (0.585, 1.039) |
| *9+* |  | 0.793*** | 1.038 | 0.837 |
|  |  | (0.714, 0.881) | (0.909, 1.184) | (0.605, 1.159) |
| Wealth index quintiles |  |  |  |  |
| *1^st^ (Poorest)* | Ref. |  |  |  |
| *2^nd^ (Poorer)* |  | 0.880*** | 1.057 | 1.085 |
|  |  | (0.817, 0.948) | (0.955, 1.170) | (0.819, 1.437) |
| *3^rd^ (Middle)* |  | 0.861*** | 1.107* | 0.932 |
|  |  | (0.791, 0.938) | (0.989, 1.239) | (0.683, 1.271) |
| *4^th^ (Richer)* |  | 0.959 | 1.318*** | 1.034 |
|  |  | (0.874, 1.052) | (1.158, 1.499) | (0.736, 1.451) |
| *5^th^ (Richest)* |  | 0.866** | 1.218** | 1.051 |
|  |  | (0.771, 0.974) | (1.043, 1.422) | (0.700, 1.577) |
| Religion |  |  |  |  |
| *Hindu* | Ref. |  |  |  |
| *Muslim* |  | 1.233*** | 1.149** | 1.161 |
|  |  | (1.140, 1.333) | (1.026, 1.286) | (0.917, 1.471) |
| *Christian* |  | 1.069 | 1.356 | 1.991*** |
|  |  | (0.854, 1.339) | (0.962, 1.913) | (1.220, 3.248) |
| *Sikh* |  | 1.263** | 0.761 | 0.593 |
|  |  | (1.051, 1.518) | (0.557, 1.040) | (0.331, 1.062) |
| *Buddhist* |  | 1.016 | 1.294 | 0.519 |
|  |  | (0.744, 1.387) | (0.787, 2.128) | (0.152, 1.772) |
| *Other* |  | 1.434** | 0.807 | 1.777 |
|  |  | (1.024, 2.007) | (0.509, 1.281) | (0.766, 4.121) |
| Caste |  |  |  |  |
| *Not backward class* | Ref. |  |  |  |
| *Scheduled caste* |  | 0.976 | 1.057 | 0.870 |
|  |  | (0.897, 1.062) | (0.942, 1.186) | (0.674, 1.124) |
| *Scheduled tribe* |  | 1.191*** | 1.086 | 1.195 |
|  |  | (1.083, 1.309) | (0.898, 1.314) | (0.873, 1.637) |
| *Other backward class* |  | 1.014 | 1.014 | 0.942 |
|  |  | (0.946, 1.087) | (0.928, 1.107) | (0.759, 1.170) |
| Residence |  |  |  |  |
| *Rural* | Ref. |  |  |  |
| *Urban* |  | 0.906*** | 0.911** | 1.008 |
|  |  | (0.843, 0.973) | (0.833, 0.997) | (0.810, 1.255) |
| Nutritional status |  |  |  |  |
| *Normal (BMI:18.5-24.9)* | Ref. |  |  |  |
| *Thin (BMI<18.5)* |  | 0.736*** | 0.970 | 0.461*** |
|  |  | (0.689, 0.787) | (0.885, 1.063) | (0.332, 0.640) |
| *Overweight (BMI: 25.0-29.9)* |  | 2.102*** | 1.551*** | 2.608*** |
|  |  | (1.962, 2.252) | (1.410, 1.707) | (2.088, 3.257) |
| *Obese (BMI≥30.0)* |  | 3.055*** | 2.857*** | 6.666*** |
|  |  | (2.746, 3.399) | (2.460, 3.320) | (5.043, 8.811) |
| No. of child born |  |  |  |  |
| *None* | Ref. |  |  |  |
| *1* |  | 0.727*** | 0.710*** | 0.612*** |
|  |  | (0.664, 0.796) | (0.635, 0.794) | (0.450, 0.834) |
| *2* |  | 0.690*** | 0.686*** | 0.476*** |
|  |  | (0.622, 0.765) | (0.598, 0.787) | (0.333, 0.680) |
| *3+* |  | 0.674*** | 0.691*** | 0.556** |
|  |  | (0.584, 0.778) | (0.568, 0.841) | (0.345, 0.896) |
| Tobacco/ alcohol consumption |  |  |  |  |
| *No* | Ref. |  |  |  |
| *Yes* |  | 1.140*** | 1.131** | 1.171 |
|  |  | (1.044, 1.246) | (1.009, 1.268) | (0.872, 1.574) |
| Oral contraception use |  |  |  |  |
| *No* | Ref. |  |  |  |
| *Yes* |  | 1.192*** | 1.150 | 1.480** |
|  |  | (1.072, 1.324) | (0.981, 1.348) | (1.059, 2.068) |
| Currently pregnant |  |  |  |  |
| *No* | Ref. |  |  |  |
| *Yes* |  | 1.229*** | 1.079** | 1.464*** |
|  |  | (1.163, 1.298) | (1.006, 1.158) | (1.221, 1.754) |
|  |  |  |  |  |
| *State Fixed Effect* |  | Yes | Yes | Yes |
|  |  |  |  |  |

Note: Estimates were obtained using complex survey weights. *** p<0.01, ** p<0.05. 95% confidence intervals are in parenthesis.
