# Supplementary material for: Stolen childhood taking a toll at young adulthood: The higher risk of high blood pressure and high blood glucose comorbidity among child brides
Source: PLOS Glob Public Health. 2022 Jun 24;2(6):e0000638. doi: 10.1371/journal.pgph.0000638 (PMC10021810; doi:10.1371/journal.pgph.0000638)
Supplement: S3 Table — Estimates were obtained using complex survey weights. ***p < 0.01, **p < 0.05. 95% confidence intervals are in parenthesis. (DOCX) [file pgph.0000638.s003.docx]

**S3 Table**. Adjusted relative risk ratios in favor of mutually exclusive high blood pressure and high blood glucose outcomes for adolescent motherhood and sociodemographic correlates

|  | **Base outcome:** | **Outcome 1:** | **Outcome 2:** | **Outcome 3:** |
| --- | --- | --- | --- | --- |
|  | Neither High  Blood Pressure  nor Blood  Glucose | High  Blood  Pressure  only | High  Blood  Glucose  only | Both High  Blood Pressure  And Blood  Glucose |
|  |  |  |  |  |
| Adolescent motherhood |  | 1.203*** | 1.067 | 1.337*** |
|  |  | (1.141, 1.267) | (0.992, 1.149) | (1.123, 1.590) |
| Age group |  |  |  |  |
| *20-22* | Ref. |  |  |  |
| *23-25* |  | 1.427*** | 1.108 | 1.489 |
|  |  | (1.295, 1.572) | (0.981, 1.253) | (0.903, 2.455) |
| *26-28* |  | 1.940*** | 1.415*** | 3.292*** |
|  |  | (1.765, 2.133) | (1.261, 1.587) | (2.116, 5.121) |
| *29-31* |  | 2.684*** | 1.802*** | 5.438*** |
|  |  | (2.448, 2.942) | (1.597, 2.034) | (3.533, 8.369) |
| *32-34* |  | 3.619*** | 2.200*** | 8.423*** |
|  |  | (3.303, 3.967) | (1.968, 2.458) | (5.493, 12.918) |
| Education |  |  |  |  |
| *No education* | Ref. |  |  |  |
| *Primary* |  | 1.005 | 0.894** | 1.038 |
|  |  | (0.932, 1.084) | (0.806, 0.992) | (0.796, 1.352) |
| *Secondary* |  | 0.926** | 0.994 | 0.879 |
|  |  | (0.867, 0.990) | (0.910, 1.086) | (0.697, 1.107) |
| *Higher* |  | 0.764*** | 0.930 | 0.744* |
|  |  | (0.678, 0.861) | (0.811, 1.068) | (0.526, 1.052) |
| Household size |  |  |  |  |
| *3 or less* | Ref. |  |  |  |
| *4-5* |  | 0.831*** | 0.994 | 0.667*** |
|  |  | (0.764, 0.903) | (0.888, 1.114) | (0.519, 0.859) |
| *6-8* |  | 0.735*** | 0.932 | 0.606*** |
|  |  | (0.675, 0.800) | (0.829, 1.047) | (0.465, 0.789) |
| *9+* |  | 0.718*** | 0.965 | 0.668*** |
|  |  | (0.649, 0.795) | (0.849, 1.097) | (0.491, 0.908) |
| Wealth index quintiles |  |  |  |  |
| *1^st^ (Poorest)* | Ref. |  |  |  |
| *2^nd^ (Poorer)* |  | 0.935 | 1.080 | 1.227 |
|  |  | (0.868, 1.006) | (0.976, 1.194) | (0.924, 1.629) |
| *3^rd^ (Middle)* |  | 0.991 | 1.181*** | 1.232 |
|  |  | (0.911, 1.077) | (1.058, 1.318) | (0.911, 1.665) |
| *4^th^ (Richer)* |  | 1.203*** | 1.487*** | 1.606*** |
|  |  | (1.100, 1.316) | (1.312, 1.685) | (1.158, 2.227) |
| *5^th^ (Richest)* |  | 1.162*** | 1.443*** | 1.845*** |
|  |  | (1.039, 1.299) | (1.247, 1.670) | (1.260, 2.702) |
| Religion |  |  |  |  |
| *Hindu* | Ref. |  |  |  |
| *Muslim* |  | 1.267*** | 1.179*** | 1.230 |
|  |  | (1.173, 1.367) | (1.055, 1.319) | (0.973, 1.556) |
| *Christian* |  | 1.080 | 1.370* | 2.085*** |
|  |  | (0.862, 1.352) | (0.977, 1.921) | (1.284, 3.387) |
| *Sikh* |  | 1.305*** | 0.789 | 0.610 |
|  |  | (1.090, 1.562) | (0.578, 1.076) | (0.340, 1.095) |
| *Buddhist* |  | 1.002 | 1.287 | 0.527 |
|  |  | (0.738, 1.361) | (0.787, 2.102) | (0.156, 1.780) |
| *Other* |  | 1.509** | 0.846 | 2.046 |
|  |  | (1.056, 2.156) | (0.544, 1.317) | (0.909, 4.605) |
| Caste |  |  |  |  |
| *Not backward class* | Ref. |  |  |  |
| *Scheduled caste* |  | 0.953 | 1.043 | 0.840 |
|  |  | (0.876, 1.036) | (0.931, 1.169) | (0.653, 1.080) |
| *Scheduled tribe* |  | 1.139*** | 1.068 | 1.106 |
|  |  | (1.037, 1.252) | (0.885, 1.289) | (0.816, 1.501) |
| *Other backward class* |  | 0.988 | 0.997 | 0.899 |
|  |  | (0.922, 1.058) | (0.912, 1.089) | (0.726, 1.113) |
| Residence |  |  |  |  |
| *Rural* | Ref. |  |  |  |
| *Urban* |  | 0.960 | 0.947 | 1.108 |
|  |  | (0.894, 1.031) | (0.867, 1.034) | (0.886, 1.385) |
|  |  |  |  |  |
| State Fixed Effect |  | Yes | Yes | Yes |
|  |  |  |  |  |

Note: Estimates were obtained using complex survey weights. *** p<0.01, ** p<0.05. 95% confidence intervals are in parenthesis.
