# Supplementary material for: Stolen childhood taking a toll at young adulthood: The higher risk of high blood pressure and high blood glucose comorbidity among child brides
Source: PLOS Glob Public Health. 2022 Jun 24;2(6):e0000638. doi: 10.1371/journal.pgph.0000638 (PMC10021810; doi:10.1371/journal.pgph.0000638)
Supplement: S4 Table — Estimates were obtained using complex survey weights. ***p < 0.01, **p < 0.05. 95% confidence intervals are in parenthesis. (DOCX) [file pgph.0000638.s004.docx]

**S4 Table**. Adjusted relative risk ratios in favor of mutually exclusive high blood pressure and high blood glucose outcomes for adolescent motherhood, sociodemographic correlates, and hypertension and diabetes risk factors

|  | **Base outcome:** | **Outcome 1:** | **Outcome 2:** | **Outcome 3:** |
| --- | --- | --- | --- | --- |
|  | Neither High  Blood Pressure  nor Blood  Glucose | High  Blood  Pressure  only | High  Blood  Glucose  only | Both High  Blood Pressure  And Blood  Glucose |
|  |  |  |  |  |
| Adolescent motherhood |  | 1.212*** | 1.103** | 1.401*** |
|  |  | (1.146, 1.283) | (1.018, 1.196) | (1.165, 1.685) |
| Age group |  |  |  |  |
| *20-22* | Ref. |  |  |  |
| *23-25* |  | 1.400*** | 1.135** | 1.459 |
|  |  | (1.268, 1.546) | (1.001, 1.288) | (0.877, 2.427) |
| *26-28* |  | 1.811*** | 1.429*** | 3.080*** |
|  |  | (1.641, 1.999) | (1.263, 1.616) | (1.952, 4.859) |
| *29-31* |  | 2.372*** | 1.764*** | 4.747*** |
|  |  | (2.144, 2.624) | (1.534, 2.027) | (3.010, 7.487) |
| *32-34* |  | 3.070*** | 2.099*** | 6.953*** |
|  |  | (2.770, 3.403) | (1.848, 2.384) | (4.401, 10.986) |
| Education |  |  |  |  |
| *No education* | Ref. |  |  |  |
| *Primary* |  | 0.970 | 0.879** | 0.961 |
|  |  | (0.899, 1.047) | (0.792, 0.975) | (0.734, 1.259) |
| *Secondary* |  | 0.869*** | 0.959 | 0.765** |
|  |  | (0.812, 0.931) | (0.878, 1.047) | (0.600, 0.976) |
| *Higher* |  | 0.712*** | 0.882 | 0.656** |
|  |  | (0.631, 0.803) | (0.767, 1.015) | (0.457, 0.941) |
| Household size |  |  |  |  |
| *3 or less* | Ref. |  |  |  |
| *4-5* |  | 0.888*** | 1.052 | 0.797 |
|  |  | (0.815, 0.967) | (0.938, 1.181) | (0.612, 1.038) |
| *6-8* |  | 0.807*** | 1.000 | 0.760 |
|  |  | (0.738, 0.882) | (0.884, 1.132) | (0.570, 1.013) |
| *9+* |  | 0.784*** | 1.034 | 0.818 |
|  |  | (0.706, 0.871) | (0.906, 1.179) | (0.591, 1.131) |
| Wealth index quintiles |  |  |  |  |
| *1^st^ (Poorest)* | Ref. |  |  |  |
| *2^nd^ (Poorer)* |  | 0.878*** | 1.055 | 1.081 |
|  |  | (0.815, 0.946) | (0.953, 1.167) | (0.816, 1.432) |
| *3^rd^ (Middle)* |  | 0.856*** | 1.103 | 0.921 |
|  |  | (0.786, 0.932) | (0.985, 1.235) | (0.674, 1.258) |
| *4^th^ (Richer)* |  | 0.949 | 1.312*** | 1.016 |
|  |  | (0.866, 1.041) | (1.153, 1.493) | (0.724, 1.425) |
| *5^th^ (Richest)* |  | 0.857*** | 1.212** | 1.031 |
|  |  | (0.763, 0.963) | (1.039, 1.415) | (0.687, 1.546) |
| Religion |  |  |  |  |
| *Hindu* | Ref. |  |  |  |
| *Muslim* |  | 1.227*** | 1.148** | 1.150 |
|  |  | (1.134, 1.327) | (1.025, 1.285) | (0.907, 1.459) |
| *Christian* |  | 1.065 | 1.356 | 1.974*** |
|  |  | (0.851, 1.334) | (0.962, 1.913) | (1.210, 3.220) |
| *Sikh* |  | 1.253** | 0.760 | 0.585 |
|  |  | (1.043, 1.505) | (0.556, 1.038) | (0.327, 1.046) |
| *Buddhist* |  | 1.009 | 1.292 | 0.510 |
|  |  | (0.739, 1.377) | (0.785, 2.125) | (0.150, 1.741) |
| *Other* |  | 1.433** | 0.808 | 1.779 |
|  |  | (1.024, 2.006) | (0.510, 1.280) | (0.766, 4.134) |
| Caste |  |  |  |  |
| *Not backward class* | Ref. |  |  |  |
| *Scheduled caste* |  | 0.975 | 1.057 | 0.870 |
|  |  | (0.896, 1.062) | (0.941, 1.186) | (0.674, 1.123) |
| *Scheduled tribe* |  | 1.187*** | 1.085 | 1.190 |
|  |  | (1.080, 1.305) | (0.897, 1.313) | (0.869, 1.631) |
| *Other backward class* |  | 1.014 | 1.013 | 0.941 |
|  |  | (0.946, 1.087) | (0.927, 1.107) | (0.758, 1.170) |
| Residence |  |  |  |  |
| *Rural* | Ref. |  |  |  |
| *Urban* |  | 0.904*** | 0.911** | 1.004 |
|  |  | (0.842, 0.971) | (0.833, 0.997) | (0.806, 1.250) |
| Nutritional status |  |  |  |  |
| *Normal (BMI:18.5-24.9)* | Ref. |  |  |  |
| *Thin (BMI<18.5)* |  | 0.737*** | 0.971 | 0.461*** |
|  |  | (0.689, 0.787) | (0.886, 1.064) | (0.332, 0.640) |
| *Overweight (BMI: 25.0-29.9)* |  | 2.105*** | 1.551*** | 2.616*** |
|  |  | (1.964, 2.256) | (1.410, 1.707) | (2.095, 3.267) |
| *Obese (BMI≥30.0)* |  | 3.061*** | 2.857*** | 6.690*** |
|  |  | (2.751, 3.405) | (2.459, 3.320) | (5.064, 8.838) |
| No. of child born |  |  |  |  |
| *None* | Ref. |  |  |  |
| *1* |  | 0.705*** | 0.696*** | 0.579*** |
|  |  | (0.643, 0.773) | (0.621, 0.781) | (0.424, 0.792) |
| *2* |  | 0.665*** | 0.666*** | 0.447*** |
|  |  | (0.597, 0.740) | (0.577, 0.770) | (0.312, 0.642) |
| *3+* |  | 0.645*** | 0.666*** | 0.519*** |
|  |  | (0.556, 0.749) | (0.543, 0.818) | (0.318, 0.845) |
| Tobacco/ alcohol consumption |  |  |  |  |
| *No* | Ref. |  |  |  |
| *Yes* |  | 1.143*** | 1.131** | 1.178 |
|  |  | (1.046, 1.248) | (1.008, 1.268) | (0.877, 1.583) |
| Oral contraception use |  |  |  |  |
| *No* | Ref. |  |  |  |
| *Yes* |  | 1.185*** | 1.146* | 1.466** |
|  |  | (1.066, 1.317) | (0.978, 1.343) | (1.048, 2.049) |
| Currently pregnant |  |  |  |  |
| *No* | Ref. |  |  |  |
| *Yes* |  | 0.468*** | 0.705*** | 0.550** |
|  |  | (0.420, 0.522) | (0.620, 0.802) | (0.319, 0.947) |
|  |  |  |  |  |
| *State Fixed Effect* |  | Yes | Yes | Yes |
|  |  |  |  |  |

Note: Estimates were obtained using complex survey weights. *** p<0.01, ** p<0.05. 95% confidence intervals are in parenthesis.
